# Risk of Kidney Dysfunction from Polypharmacy among Older Patients: A Nested Case-Control Study of the South Korean Senior Cohort

**DOI:** 10.1038/s41598-019-46849-7

**Published:** 2019-07-18

**Authors:** Hyeonjin Kang, Song Hee Hong

**Affiliations:** 1 College of Pharmacy, Seoul National University, Seoul, Korea; 2 Research Institute of Pharmaceutical Science, Seoul National University, Seoul, Korea

**Keywords:** Epidemiology, Risk factors

## Abstract

Polypharmacy, the concurrent use of multiple medicines, could increase the risk of kidney dysfunction among older adults because it likely burdens the aging kidneys to excrete multiple pharmaceutical ingredients and their metabolites. This study aimed to examine the relation between polypharmacy and kidney dysfunction among older patients. A nested case-control study was conducted using the National Health Insurance Service – Senior Cohort (NHIS-SC, 2009–2013), representative of the Korean senior population. It consisted of all health insurance claims linked to records of mandatory health examination. Kidney dysfunction was defined as having an eGFR lower than 60, with a decline rate of 10% or more compared to the baseline eGFR. Polypharmacy was defined based on daily counts of pharmaceutical ingredients during one year prior to the case’s event date. It was classified into polypharmacy (five to 10 ingredients) and excessive polypharmacy (10 or more ingredients). After matching case and control groups based on a range of potential confounders, conditional logistic regression was performed incorporating adjustments on disease-specific, medication-specific, and lifestyle-related risk factors. The matching resulted in 14,577 pairs of cases and controls. Exposure to polypharmacy was significantly associated with increase in the risk of kidney dysfunction; i.e., crude model (polypharmacy: OR = 1.572, 95% CI = 1.492–1.656; excessive polypharmacy: OR = 2.069, 95% CI = 1.876–2.283) and risk adjustment model (polypharmacy: OR = 1.213, 95% CI = 1.139–1.292; excessive polypharmacy: OR = 1.461, 95% CI = 1.303–1.639). The significant associations were robust across different definitions of kidney dysfunction. These findings inform healthcare providers and policy makers of the importance of polypharmacy prevention to protect older adults from kidney dysfunction.

## Introduction

The issue of polypharmacy, the concomitant use of multiple medicines, has been around for quite some time. While a consistent definition has yet to evolve^1–3^, its prevalence has been increasing rapidly over time^4–6^. Especially among older adults, it is quite prevalent not only in Korea^7–9^ but also in other countries^5,6,10^, as aging increases disease morbidity, which makes polypharmacy more prevalent among older adults^11–13^. Older adults, thus, would be at greater risk of adverse drug reactions, not only from polypharmacy but also from their weakening physiological functions, such as those of the kidneys^1,14–18^.

Polypharmacy could seriously damage the kidney because the former likely burdens the latter to excrete a wide range of drugs and their metabolites^18,19^. While many studies have examined the negative effect of polypharmacy on various health outcomes, such as falls^20–23^, fractures^24^, delirium^25^, dementia^8,26^, and Parkinsonism^27^ among older adults, only a few have investigated the association between polypharmacy and kidney function, out of which two examined the risk of acute renal failure^28,29^ and two examined chronic kidney diseases (CKD)^30,31^. However, the two studies on CKD reported inconsistent results since they used different approaches to risk adjustment for different operational definitions of CKD. Furthermore, the study results were susceptible to bias from cross-sectional data. Therefore, there is a critical need to generate more scientific evidence on the temporal relation between exposure to polypharmacy and risk of kidney dysfunction.

This study aimed to document the temporal relationship between polypharmacy and kidney dysfunction among old patients in Korea, the population of which is aging at an unprecedentedly rapid rate. Furthermore, the study further aimed to determine the significant association after risk adjustments on disease-specific, medication-specific, and lifestyle-related risk factors.

## Methods

### Study population

We used the population-based cohort of National Health Insurance Service–Senior Cohort (NHIS-SC) data established by the National Health Insurance Service (NHIS), the single payer of South Korea. Accordingly, the NHIS collects information on eligibility, health insurance claims, and lab values from mandatory periodic health check-ups for selected populations. During the health check-ups, information on lifestyles is collected through interviews. Moreover, the NHIS-SC was constructed based on a 10% random sample (n = 558,147) of the population aged 60 or over as of December 2002. The sample cohort was tracked for 11 years until 2013. Consequently, this study was approved by the Seoul National University Institutional Review Board (IRB No. E1801/001-001). Additionally, obtaining informed consent from the study population was waived because this study involves an analysis of existing data. All methods were performed in accordance with the approved guideline and regulation. Furthermore, our study used the NHIS-SC data from 2009 to 2013 since the key variable of serum creatinine (SCr), an indicator of kidney function, became available in 2009.

In this study, patients aged 65–84 who had a normal range of serum creatinine (SCr of 0.5–1.5) and a normal value of estimated glomerular filtration rate (eGFR of 60 or higher) at a baseline health check-up were included. In Korea, older adults are typically defined as those with ages 65 years or older to qualify for a range of senior-specific benefits. As the NHIS cohort got older, the age group of 60–64 no longer existed during the study period. Thus, the youngest age group in the study was between the ages of 65–69. The oldest age group (85 or older) was excluded because they are known to be quite different from the younger groups. Furthermore, patients who had not had their next health check-up within three years from the baseline date (n = 87,147) were excluded. Out of the aforementioned, patients with outlier values of SCr at the last check-up as well as patients with a history of renal disease prior to the case’s event date were excluded.

### Study design

**Figure 1:**
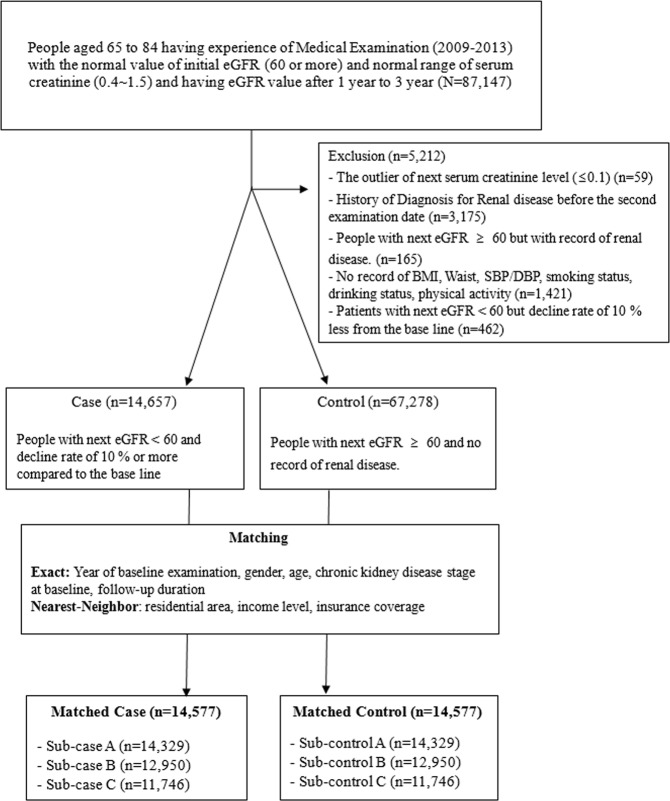
Flowcharts for study subjects.

A nested case-control study was designed, with cases of patients who had developed kidney dysfunction during the follow-up health check-up some time in 2013. Kidney dysfunction was defined as a follow-up eGFR lower than 60 with a decline rate of 10% or more from the initial eGFR. Controls were those patients without renal disease diagnoses who had normal eGFR at initial and follow-up check-ups. After the exclusion of patients without health check-up information, cases (n = 14,657) and controls (n = 67,278) were matched 1:1 based on a wide range of covariates, excluding risk factors. The matching was exact in the year of baseline examination, gender, age, chronic kidney disease stage at baseline, and follow-up duration, but the nearest neighbor in resident area, medical insurance coverage, and income level. After matching, the numbers of the final sample were 14,577 each for case and control group (Refer to Fig. 1).

### Kidney dysfunction

Glomerular filtration rate (GFR) is regarded as the best indicator for kidney function, and is the reference criterion for classification of kidney disease established by the National Kidney Foundation’s Kidney Disease Outcomes Quality Initiative^32,33^. The cut-off point of 60 for GFR indicates chronic kidney disease^32^. Kidney dysfunction is operationally defined as the status of having GFR lower than 60 ml/min/1.73 m^2^, while having a decline rate of 10% or more from baseline GFR. The decline rate of 10% or more was to exclude patients with little difference in GFR between two health check-ups. In addition, the literature reports that annual decline rate can predict kidney disease progress^34^. Accordingly, annual eGFR decline rates of 3, 4, and 5 ml/min/1.73 m^2^/year were also used when defining kidney dysfunction in separate subgroup analyses.

It is common to use the eGFR from SCr concentration^35^. This study used the Chronic Kidney Disease Epidemiology Collaboration (CKD-EPI) equation to estimate GFR. It is preferred for estimating GFR in adults because of its accuracy^36,37^.

### Polypharmacy

Polypharmacy was computed based on the daily counts of pharmaceutical ingredients of all prescription drugs taken during 1 year prior to the case’s event date and subsequently classified into non-polypharmacy (daily use of less than five), polypharmacy (daily use of five to less than 10), and excessive polypharmacy (daily use of 10 or more). Additionally, the prescription drugs included all drugs from dental to medical care as well as inpatient and outpatient care. Furthermore, they included fixed-dose combination drugs. However, when counting the number of pharmaceutical ingredients, digestives and fillers were excluded along with OTC drugs, traditional medicines, and drugs absent in the NHIS formulary. Despite the exclusion, underestimation of polypharmacy is not much of an issue because the NHIS formulary is comprehensive; i.e., it only excludes some of the new drugs that do not meet the cost-effectiveness criteria.

$${\rm{Daily}}\,{\rm{use}}\,{\rm{counts}}\,{\rm{of}}\,{\rm{pharmaceutical}}\,{\rm{ingredients}}\,{\rm{per}}\,{\rm{year}}=\frac{\sum {K}_{i}\ast {days}\,{of}\,{{supply}}_{{i}}}{1\,{year}}$$

where: *Ki* is the number of active pharmaceutical ingredients of a prescription drug *i*.

### Risk adjustment

A wide range of well-known kidney dysfunction risk factors were identified from the literature and classified into disease-specific, medication-specific, and lifestyle-related risk factors. The disease-specific risk factors included were hypertension (HTN)^38–49^, diabetes mellitus (DM)^38–43,46,50^, congestive heart failure (CHF)^42,51–53^, ischemic heart disease (IHD)^42^, arrhythmia^42^, gout^54^, hypercholesterolemia (Hyper-TC)^41,42,55^, hypertriglyceridemia (Hyper-TG)^41–43,55,56^, lower high density lipoprotein cholesterol (Lower-HDL-C)^41–43,55,57^, higher low density lipoprotein cholesterol (Higher-LDL-C)^42,55,57^, and obesity^38,40,50,58–62^. The medication-specific risk factors were angiotensin-converting-enzyme inhibitors (ACEIs)^63–66^, angiotensin II receptor blockers (ARBs)^63–67^, metformin^68^, statins^69^, non-steroidal anti-inflammatory drugs (NSAIDs)^50,63–66,70,71^, proton pump inhibitors (PPIs)^64,65,72–74^, and allopurinol^63,65,75^. Finally, the lifestyle-related risk factors were smoking^38,39,41–43,50,59,76–79^, alcohol consumption^41,43,76,80,81^, and physical activity^43,59,82–85^.

Disease-specific risk factors were mainly determined based on the diagnosis code, the 10^th^ revision of the International Statistical Classification of Diseases and Related Health Problems (ICD-10), whether people had had the relevant disease code or not from the baseline to the case’s event date: HTN (I10-I15); DM (E10-E14); CHF (I50); IHD (I20-I25); arrhythmia (I49); and gout (M10). Obesity was based on Body Mass Index (BMI), and classified into underweight (less than 18.5), normal weight (18.5–22.9), overweight (23.0–24.9), and obese (more than 25), according to the Asia Pacific regional guidelines of the World Health Organization and International Obesity Task Force. All definitions related to lipid status were based on fasting lipid measure. Hyper-TC was defined as total cholesterol level more than 240 mg/dL; Lower HDL-C as HDL-C ≤ 40 mg/dL; Higher LDL-C as LDL-C ≥ 140 mg/dL; and Hyper-TG as triglycerides ≥ 150 mg/dL.

Exposure to each medication risk factor was defined depending on types of medication. First, exposures to chronic medicines (ACEIs, ARBs, Metformin, Statins) were defined based on a PDC (Proportion of Days Covered) of 50% or higher during one year prior to the case’s event date. Second, exposures to NSAIDs and PPIs were defined the same way as above using 90 days instead of one year. Third, exposure to allopurinol was defined based on a record of prescription two weeks prior to the event date.

Subsequently, smoking status was classified as smoker or non-smoker based on consecutive non-smoker responses at baseline as well as follow-up health check-ups to a question about whether a patient had smoked more than 5 boxes or 100 cigarettes in their lifespan. On the other hand, alcohol consumption status was defined based on the mean number of drinking days per week (non-drinker: 0–1 day per week) for the responses at baseline and follow-up. The exercise status was also defined based on the mean number of exercise days per week for the responses at baseline and follow-up, in which each patient performed moderate physical activity for at least 30 minutes (non-exerciser: 0–1 day per week).

### Sample size and power computation

This study is a retrospective case-control study based on a 10% random sample cohort of Korean seniors. Thus, we took the approach of computing power from the number of patients who met our inclusion/exclusion criteria rather than figuring out the sample size that achieves, at least, the power level of 80% given an effect size of OR = 1.2 from a logistic regression model involving 15 covariates. Consequently, the computed power well exceeded 80% given the number of case-control pairs of 14,577.

### Statistical analysis

Baseline characteristics of the cases and controls were compared using a t-test for continuous variables and chi-square test for categorical variables. Conditional logistic regression was used to calculate the odds ratio (OR) and its 95% confidence intervals (CIs). Risk factors were adjusted step by step: First, the disease and lifestyle risk factors were included in the adjusted model. Second, only exposure to medication-related factors was considered. Third, as the final model in this study, all risk factors were included. Subgroup analyses were conducted incorporating different definitions of kidney dysfunction into the final model.

## Results

### Description of study sample

From the cases (n = 14,657) and controls (n = 67,278) that met the inclusion/exclusion criteria in the cohort, the matching resulted in 14,577 pairs of cases and controls. Before matching, cases and controls were all different in each matching variable, except for insurance type. However, after matching, cases and controls were well-balanced with no practical differences. In fact, cases and controls were exactly the same, except for income and residential areas (Refer to details in Supplementary Table S1).

This study did not match cases and controls on risk factors, but instead included those risk factors later for risk adjustment models. As seen in the study sample description, exposures to polypharmacy and excessive polypharmacy were higher among cases than among controls: 33.15% vs. 25.23% respectively for polypharmacy and 8.49% vs. 4.98% for excessive polypharmacy. Exposures to all other risk factors were also significantly higher among cases than controls except for Hyper-TC, Higher-LDL-C, smoking, and physical activity (Table 1).

**Table 1:** Description of Study Sample.

		Matched Case (n = 14,577)		Matched Control (n = 14,577)		p-value
		Freq.	(%)	Freq.	(%)	
Polypharmacy (PP)						
Non-PP		8507	58.36	10173	69.79	<0.0001
PP		4832	33.15	3678	25.23	
E-PP		1238	8.49	726	4.98	
**Disease-specific**						
HTN		9913	68.00	8245	56.56	<0.0001
DM		3856	26.45	2941	20.18	<0.0001
CHF		623	4.27	364	2.50	<0.0001
IHD		2105	14.44	1541	10.57	<0.0001
Arrhythmia		271	1.86	192	1.32	0.0002
Gout		367	2.52	168	1.15	<0.0001
Obesity level	Underweight	414	2.84	635	4.36	<0.0001
	Normal weight	4817	33.05	5453	37.41	
	Overweight	3840	26.34	3773	25.88	
	Obese	5506	37.77	4716	32.35	
Hyper-TC (240≤)		1977	13.56	1907	13.08	0.2280
Hyper-TG (150≤)		5303	36.38	4488	30.79	<0.0001
Lower-HDL-C (<40)		2335	16.02	1878	12.88	<0.0001
Higher-LDL-C (140≤)		3566	24.46	3577	24.54	0.8810
**Medication-specific**						
ACEIs		664	4.56	451	3.09	<0.0001
ARBs		5401	37.05	3615	24.80	<0.0001
Metformin		2168	14.87	1590	10.91	<0.0001
Statins		3301	22.65	2619	17.97	<0.0001
NSAIDs		2641	18.12	2294	15.74	<0.0001
PPIs		885	6.07	699	4.80	<0.0001
Allopurinol		62	0.43	25	0.17	<0.0001
**Lifestyle-related**						
Smoking		4705	32.28	4599	31.55	0.1829
Drinking		4075	27.95	4220	28.95	0.0600
Physical activity		5637	38.67	5597	38.40	0.6300

Non-PP: Non-polypharmacy, use of less than five drugs; PP: Polypharmacy, use of five to 10 drugs; E-PP: Excessive polypharmacy, use of 10 or more drugs.

HTN: hypertension; DM: diabetes mellitus; CHF: congestive heart failure; IHD: ischemic heart disease;

Underweight: BMI < 18.5; Normal: BMI < 23; Overweight: BMI < 25; Obese: BMI ≥ 25.

ACEIs: Angiotensin-Converting-Enzyme Inhibitors; ARBs: Angiotensin II Receptor Blockers; NSAIDs: Non-Steroidal Anti-Inflammatory Drugs; PPIs: Proton Pump Inhibitors.

### Risk of kidney dysfunction from polypharmacy

Compared to controls without kidney dysfunction, cases with kidney dysfunction were significantly associated with higher odds of exposure to polypharmacy, not only in the crude conditional logistic regression model (polypharmacy: Crude OR = 1.572, 95% CI = 1.492–1.656; excessive polypharmacy: Crude OR = 2.069, 95% CI = 1.876–2.283) but also in each risk-adjusted model. In Model 1, adjusted for the disease-specific and lifestyle-related risk factors, Adj. OR was 1.287 (95% CI = 1.212–1.366) for polypharmacy and 1.603 (95% CI = 1.439–1.787) for excessive polypharmacy. The significant associations were also present for the other two risk adjustment models (Table 2).

**Table 2:** Associative Risk Factors for Kidney Dysfunction.

	Unadjusted			Adjusted								
				Model 1			Model 2			Model 3		
	OR	95% CI		OR	95% CI		OR	95% CI		OR	95% CI	
**Polypharmacy**												
PP (Ref = Non-PP)	1.572	1.492	1.656	1.287	1.212	1.366	1.301	1.225	1.380	1.213	1.139	1.292
E-PP (Ref = Non-PP)	2.069	1.876	2.283	1.603	1.439	1.787	1.589	1.424	1.772	1.461	1.303	1.639
**Disease-specific**												
Hypertension	—	—	—	1.336	1.265	1.412	—	—	—	1.141	1.073	1.213
Diabetes	—	—	—	1.122	1.056	1.193	—	—	—	1.107	1.021	1.200
CHF	—	—	—	1.361	1.186	1.562	—	—	—	1.329	1.156	1.527
IHD	—	—	—	1.073	0.993	1.160	—	—	—	1.066	0.984	1.154
Arrhythmia	—	—	—	1.130	0.928	1.377	—	—	—	1.110	0.910	1.354
Gout	—	—	—	1.912	1.575	2.321	—	—	—	1.853	1.507	2.277
Normal-weight (Ref = Under-)	—	—	—	1.225	1.069	1.405	—	—	—	1.216	1.060	1.396
Over-weight (Ref = Under-)	—	—	—	1.304	1.133	1.501	—	—	—	1.281	1.112	1.475
Obese (Ref = Under-)	—	—	—	1.410	1.227	1.621	—	—	—	1.377	1.197	1.584
Hyper-TG	—	—	—	1.171	1.111	1.235	—	—	—	1.171	1.111	1.235
Lower-HDL-C	—	—	—	1.169	1.090	1.254	—	—	—	1.171	1.091	1.257
Hyper-LDL-C	—	—	—	1.000	1.000	1.001	—	—	—	1.001	1.000	1.001
**Medication-specific**												
ACEI	—	—	—	—	—	—	1.444	1.273	1.637	1.348	1.183	1.536
ARB	—	—	—	—	—	—	1.594	1.508	1.685	1.449	1.361	1.543
Metformin	—	—	—	—	—	—	1.089	1.010	1.174	0.989	0.894	1.094
Statins	—	—	—	—	—	—	1.021	0.957	1.088	0.973	0.910	1.040
NSAIDs	—	—	—	—	—	—	1.052	0.986	1.122	1.039	0.972	1.110
PPI	—	—	—	—	—	—	1.083	0.974	1.205	1.092	0.979	1.217
Allopurinol	—	—	—	—	—	—	2.007	1.249	3.226	1.180	0.692	2.013
**Lifestyle-related**												
Smoking	—	—	—	1.071	0.998	1.149	—	—	—	1.075	1.001	1.154
Drinking	—	—	—	0.967	0.908	1.029	—	—	—	0.962	0.903	1.024
Physical activity	—	—	—	1.017	0.966	1.070	—	—	—	1.016	0.965	1.069

Non-PP: Non-polypharmacy for daily counts of less than 5 drugs per year; PP: Polypharmacy for daily counts of 5–10 drugs per year; E-PP: Excessive polypharmacy for daily counts of 10 or more drugs per year.

HTN: hypertension; DM: diabetes mellitus; CHF: congestive heart failure; IHD: ischemic heart disease;

Underweight: BMI < 18.5; Normal: BMI < 23; Overweight: BMI < 25; Obese: BMI ≥ 25.

ACEIs: Angiotensin-Converting-Enzyme Inhibitors; ARBs: Angiotensin II Receptor Blockers; NSAIDs: Non-Steroidal Anti-Inflammatory Drugs; PPIs: Proton Pump Inhibitors.

Significantly positive associations between exposure to each risk factor and kidney dysfunction were observed for all disease-related risk factors except for IHD and arrhythmia; in Model 3 that was adjusted for all risk factors, IHD and arrhythmia respectively had Adj. OR of 1.066 (95% CI = 0.984–1.154) and of 1.110 (95% CI = 0.910–1.354). As for the medication-specific risk factors, only two medications had significant associations with kidney dysfunction; Adj. OR for ACEI was 1.348 (95% CI: 1.183–1.536), for ARB 1.449 (95% CI: 1.361–1.543). Metformin and allopurinol had a significant association in Model 2, but no longer when all other risk factors were adjusted.

Finally, for the lifestyle-related risk factors, overweight people were more likely to have kidney dysfunction (normal weight: Adj. OR = 1.216, 95% CI: 1.060–1.396; overweight: Adj. OR = 1.281, 95% CI: 1.112–1.475; obese: Adj. OR = 1.377, 95% CI: 1.197–1.584, ref = underweight). For lipid measures, Hyper-TG and Lower-HDL-C were significantly associated with kidney dysfunction (Hyper-TG: Adj. OR = 1.171, 95% CI: 1.111–1.235; Lower-HDL-C: Adj. OR = 1.171, 95% CI: 1.091–1.257; Higher-LDL-C: Adj. OR = 1.000, 95% CI: 1.000–1.001). As for smoking, drinking, and physical activity, only smoking was significantly associated with kidney dysfunction (Adj. OR: 1.075, 95% CI: 1.001–1.154).

### Subgroup analyses for different definitions of kidney dysfunction

Study findings would be robust when they were consistent across different definitions of kidney dysfunction. We generated several subgroups of cases and controls using different definitions. In the original model, we defined kidney dysfunction using eGFR lower than 60 with a decline of 10% or more compared to baseline eGFR. We constructed subgroups using annual rather than total decline rate. Sub-case A was defined as patients with next eGFR less than 60 with an annual decline rate of 3 ml/min/1.73 m^2^ or more. The matched controls were sub-control A (each N = 14,329). Sub-case/control B (each N = 12,950) and sub-case/control C (each N = 11,746) were defined using the annual decline rates of 4 ml/min/1.73 m^2^ or more and 5 ml/min/1.73 m^2^ or more respectively.

**Figure 2:**
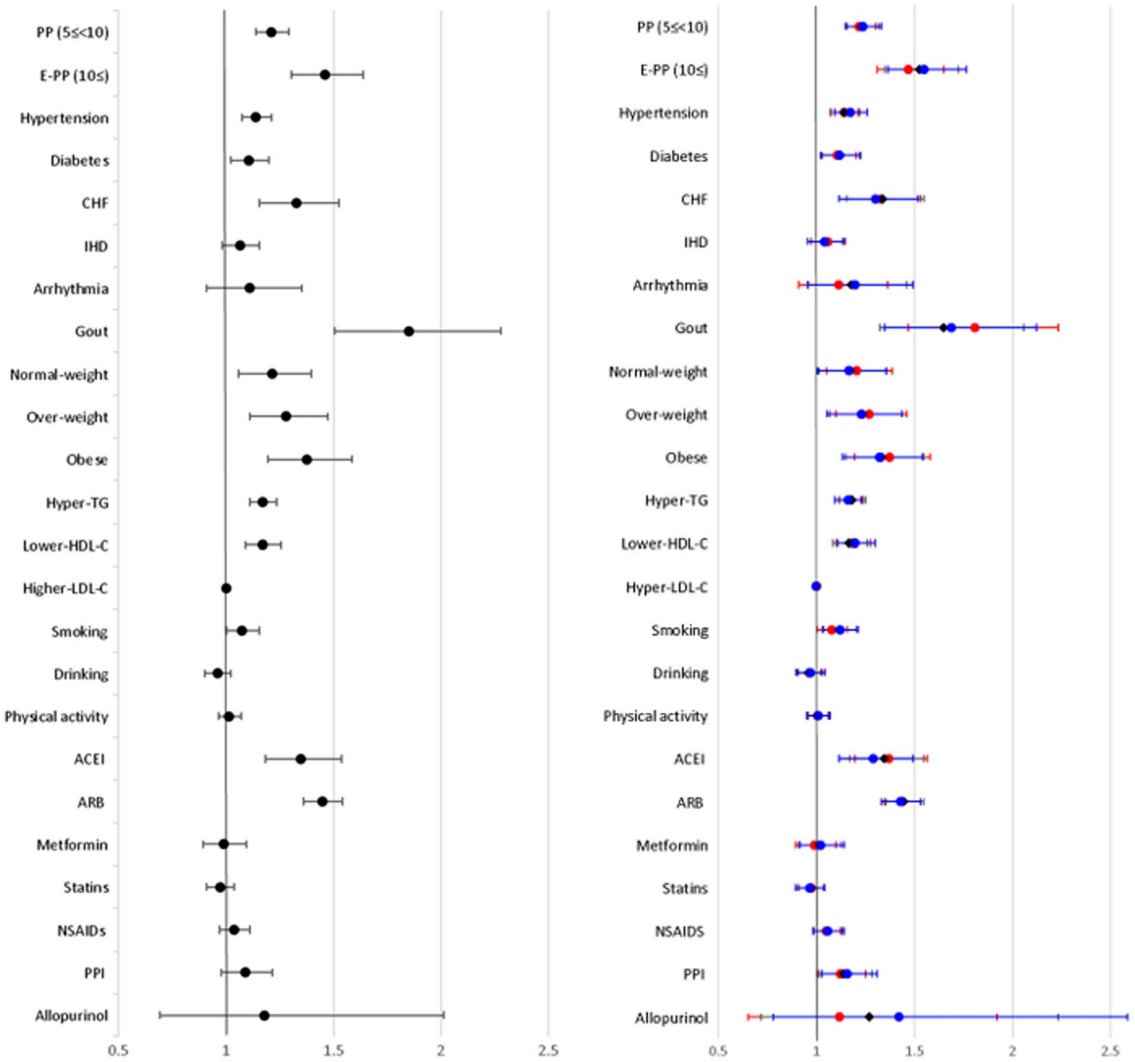
Odd ratios of exposures to each risk factor and kidney dysfunction for different Operationalisations of kidney dysfunction. (Left: Results from the main analysis; Right: Results from the subgroup analyses: red dot for subgroup A; black diamond for subgroup B; and blue dot for subgroup C). PP: Polypharmacy, use of five to 10 drugs; E– PP: Excessive polypharmacy, use of 10 or more drugs; HTN: hypertension; DM: diabetes mellitus; CHF: congestive heart failure; IHD: ischemic heart disease; Underweight: BMI < 18.5; Normal: BMI < 23; Overweight: BMI < 25; Obese: BMI ≥ 25; ACEIs: Angiotensin-Converting-Enzyme Inhibitors; ARBs: Angiotensin II Receptor Blockers; NSAIDs: Non-Steroidal Anti-Inflammatory Drugs; PPIs: Proton Pump Inhibitors.

Across different subgroups, polypharmacy and excessive polypharmacy were significantly associated with kidney dysfunction: respective crude ORs were 1.572 and 2.069 for subgroup A; 1.574 and 2.095 for subgroup B; and 1.624 and 2.216 for subgroup C (Fig. 2 and Supplementary Table S2). In addition, all the adjusted ORs of polypharmacy and excessive polypharmacy were significant: 1.220 and 1.468 for subgroup A; 1.232 and 1.525 for subgroup B; and 1.240 and 1.551 for subgroup C, respectively. All other risk factors had the same significant association with kidney dysfunction to the original analysis, except that the use of PPI was also significantly associated with kidney dysfunction, with Adj. OR of 1.212 (95% CI: 1.004–1.251) for subgroup A, 1.140 (95% CI: 1.016–1.280) for subgroup B, and 1.160 (95% CI: 1.027–1.311) for subgroup C. (Fig. 2. Refer to details in Supplementary Table S2)

## Discussion

Compared to controls who had kept their normal kidney function since the baseline examination, the matched cases with kidney dysfunction had a higher exposure to polypharmacy as well as to excessive polypharmacy (polypharmacy: 33.15% vs. 25.23: excessive polypharmacy: 8.49% vs. 4.98%). The higher exposure to polypharmacy among cases of kidney dysfunction than among controls with normal kidney function was also observed in the crude conditional logistic regression (polypharmacy: OR = 1.572, 95%CI = 1.492–1.656; excessive polypharmacy: OR = 2.069, 95% CI = 1.876–2.283), as well as in the risk adjusted model (polypharmacy: Adj. OR = 1.213, 95% CI = 1.139–1.292; excessive polypharmacy: Adj.OR = 1.461, 95% CI = 1.303–1.639). These results are consistent with the findings of three previous studies^28–30^. Konig *et al*.^30^ reported a crude OR of 2.07 (95% CI: 1.54–2.74) and an adjusted OR of 1.54 (95% CI: 1.14–2.08) based on the Berlin Aging Study II (BASE –II) cohort. The other two studies also reported a significant association between polypharmacy and kidney dysfunction, while using different concepts of polypharmacy (duration of polypharmacy, polypharmacy of cardiovascular medicines) and focusing on kidney dysfunction of acute renal failure and injury. However, our study results were not consistent with Sutaria *et al*.’s study^31^. Their study found a negative effect of polypharmacy on CKD based on an unadjusted model, but a protective, though not statistically significant, effect of polypharmacy on CKD when adjusting for age, cardiovascular disease, and diabetes mellitus. There are some differences between the studies. Firstly, their study is a cross-sectional, while ours is a nested case control. Their study did not control for the other important risk factors such as those lifestyle-related and medication-specific. Furthermore, the effect of polypharmacy might be masked by the large variations of age effect, which was matched between cases and controls in our study. In other words, our study focused on risk factors for kidney dysfunction after exactly matching other covariates between cases and controls.

Moreover, this study confirmed that most of the risk factors known to impair kidney function are associated with kidney dysfunction (Table 2). However, interestingly, ACEIs/ARBs^50,63–67,70^ were found to be significantly associated with kidney dysfunction despite the fact that they are recommended as first-line hypertension therapy for patients with a compelling condition of CKD^86–89^ due to their reno-protective effect. Although it is initially tempting to claim that the adverse associations just reflect the fact that patients who take ACEI/ARB are likely to have had kidney dysfunction, the associations from our case-control study are from a temporal sequence where exposure to ACEI/ARB precedes the outcome occurrence of kidney dysfunction. On the other hand, it is plausible that patients who take ACEI/ARB are likely to have had conditions of HTN/DM and are thus more likely to develop kidney dysfunction, not from the medications but from the diseases. Recognizing the potential confounding effects of HTN/DM, our study did control for presence of the diseases in the risk adjustment model to separate the disease- and medication-specific effects. As the result, our study found that risk of kidney dysfunction was associated not only with presence of HTN/DM but also with exposure to ACEI/ARB. Our study is not the first to report that use of ACEI/ARB may not always be reno-protective, especially in real world settings.^90–92^

The other risk factors known to damage kidneys but not found significant in this study are LDL, drinking, Proton Pump Inhibitors (PPIs), Metformin, Statin, and NSAIDs. However, plenty of evidence supports our study. Moreover, LDL is the least likely risk factor for kidney dysfunction among cholesterol types^50,57^. In addition, drinking, especially moderate alcohol consumption, has no adverse effect on kidney function^93^. Accordingly, PPIs have the weakest level of evidence for being risk factors for kidney dysfunction^74^. In our study, PPIs were not significantly associated with kidney dysfunction in the main analysis but were significantly associated in the subgroup analysis. Furthermore, some evidence in the literature suggests that Metformin^94,95^ and statin^96^ are not associated with kidney function. Despite the widely-known adverse association of NSAIDs with kidney dysfunction^50,63–66,70^, our study reports no such association. Therefore, perhaps the contradictory findings may have resulted from two different study populations. While our study findings come from patients with normal kidney functioning, the findings in the literature are based on patients with kidney dysfunction. Alternatively, it is likely that the duration of NSAID exposure could affect the occurrence of reno-toxicity. Our study did not stratify NSAID exposure into long-term vs. short-term. Instead, we dichotomized NSAID exposure based on a proportion of days covered (PDC) of 50% or higher for 90 days prior to the kidney dysfunction. While our study is not the first to report the contradictory finding^97,98^ future studies need to examine whether the risk of reno-toxicity depends on long-term vs. short-term exposure to NSAID. Additionally, allopurinol is known to be reno-protective for hyperuricemia^75^, but can also be reno-toxic for interstitial nephritis^50,63,65,66,70^. In our study, allopurinol had an adverse effect on the kidneys when not adjusted for gout but lacked an adverse effect when adjusted for gout. As a result, more studies are required in order to understand this phenomenon as reflected in the comments of Stamp *et al*.^99^.

A key strength of this study is the use of linked data of health check-up information with prescription claims, which was constructed as cohort data under the universal health coverage system in Korea. Although various studies with slightly different operational definitions have examined the association between polypharmacy and kidney disease, they all had limitations, in that they didn’t consider obesity and smoking, which are important risk factors for kidney function^28,29,31^, or were biased due to using a self-reported questionnaire^30^. Consequently, considering the information available from our data, the risk factors for kidney disease considered and reflected in the study model are more comprehensive than in other studies. Moreover, despite adjusting for various covariates and/or risk factors, we have identified the associative risk of polypharmacy for kidney dysfunction. In addition, factors identified associated with kidney dysfunction, apart from exposure to polypharmacy, are hypertension, diabetes, congestive heart failure, gout, obesity, hyper-TG, lower-HDL-C, smoking, and use of ACEIs/ARBs (as well as the use of PPIs in a more rigorous sub-group). Despite past studies having identified associative risks for kidney disease, medicines taken by patients, which are one of the factors that burden kidney function, were not considered in those studies. These medicines not only include polypharmacy-related exposure, but also other medicines known for being associated with the kidneys, such as ACEIs/ARBs, Metformin, Statins, NSAIDs, PPIs, and Allopurinol.

However, there are limitations in our study. Firstly, other medicines with potential nephrotoxicity were not considered in the statistical model due to the limited number of their takers and little difference between case and control groups resulting from basic statistical analysis. Such drugs include osmotic agents, contrast, methotrexate, calcineurin inhibitors, and certain antibiotics (refer to Supplementary Table S3). Secondly, only prescribed medicines in the NHIS formulary are included while accounting for polypharmacy. Consequently, not considering the use of non-formulary drugs, drug samples, over-the-counter (OTC) drugs, supplements, and vitamins leads to underestimating polypharmacy and a restricted interpretation of the association between polypharmacy and kidney dysfunction. Thirdly, confounders might be associated with kidney function despite adjusting more covariates than before. For example, parenteral medicines and other diseases were not accounted for.

In conclusion, this study found that exposure to polypharmacy was significantly associated with increases in the risk of kidney dysfunction among older patients. There was a temporal association for different risk adjustments as well as for different subgroup analyses. These findings inform healthcare providers and policy makers of the importance of polypharmacy prevention to protect older adults from kidney dysfunction.

## Supplementary information

Supplementary Tables

## Data Availability

The dataset generated and analysed in this study are available from the corresponding author on reasonable requests.
