# Supplementary material for: Risk of Kidney Dysfunction from Polypharmacy among Older Patients: A Nested Case-Control Study of the South Korean Senior Cohort
Source: Sci Rep. 2019 Jul 18;9:10440. doi: 10.1038/s41598-019-46849-7 (PMC6639333; doi:10.1038/s41598-019-46849-7)
Supplement: Supplementary file 1 — Supplementary Tables [file 41598_2019_46849_MOESM1_ESM.pdf]

Risk of Kidney Dysfunction from Polypharmacy among Older Patients: A Nested Case-Control Study of the South Korean Senior Cohort

Hyeonjin Kang[1,2], Song Hee Hong[1,2*]

[1] College of Pharmacy, Seoul National University, Seoul, Korea

[2] Research Institute of Pharmaceutical Science, Seoul National University, Seoul, Korea

*** Corresponding author**

E-mail: songhhong@snu.ac.kr

Supplementary Table S1.

Basic statistics for matched variables before and after matching

	Unmatched					Matched				
	Case (n=14,657)		Control (n=67,257)		p	Matched Case (n=14,577)		Matched Control (n=14,577)		p
	Freq.	(%)	Freq.	(%)		Freq.	(%)	Freq.	(%)	
**Year of Baseline examination**										
2009	7300	49.81	15406	22.91	<.0001	7240	49.67	7240	49.67	1
2010	6113	41.71	44392	66.00		6100	41.85	6100	41.85	
2011	1198	8.17	7217	10.73		1194	8.19	1194	8.19	
2012	46	0.31	242	0.36		43	0.29	43	0.29	
**Initial Kidney Function**										
eGFR ≥90	1051	7.17	11865	17.64	<.0001	1044	7.16	1044	7.16	1
eGFR ≥60	13606	92.83	55392	82.36		13533	92.84	13533	92.84	
**Follow-up duration (year)**										
1≤ < 2	7482	51.05	32333	48.07	<.0001	7420	50.90	7420	50.90	1
2≤ ≤ 3	7175	48.95	34924	51.93		7157	49.10	7157	49.10	
**Gender**										
Female	8282	56.51	36344	54.04	<.0001	8209	56.31	8209	56.31	1
Male	6375	43.49	30913	45.96		6368	43.69	6368	43.69	
**Age (year)**										
65 ≤ < 75	10244	69.89	52998	78.80	<.0001	10237	70.23	10237	70.23	1
75 ≤ < 85	4413	30.11	14259	21.20		4340	29.77	4340	29.77	
**Coverage**										
Health Insurance	14645	99.92	67221	99.95	0.1988	14577	100.00	14577	100.00	1
Medical Aids	12	0.08	36	0.05			0.00		0.00	
**Income level[1]**										
High	8380	57.17	39955	59.41	<.0001	8337	57.19	8448	57.95	0.405
Medium	2999	20.46	13660	20.31		2988	20.50	2950	20.24	
Low	3278	22.36	13642	20.28		3252	22.31	3179	21.81	
**Residential area[2]**										
Metrocity	5384	36.73	23061	34.29	<.0001	5373	36.86	5383	36.93	0.559
City	3293	22.47	14426	21.45		3287	22.55	3214	22.05	
Others	5980	40.80	29770	44.26		5917	40.59	5980	41.02	

[1]Income level is classified based on the household income deciles in the national health insurance eligibility file. Its respective category of High, Medium, or Low represents the top 3, next top 3, or bottom 4 deciles of the household income

[2]Residential area is classified into the three groups considering administrative region and city population size: Metrocity (Seoul and Kyeonggi-do); City (Busan, Daegu, Incheon, Gwangju, Daejeon, Ulsan); and Others (Other region)

Supplementary Table S2.

Subgroup Analysis for Associated Risk Factors for Kidney Dysfunction

	**Subgroup A**			**Subgroup B**			**Subgroup C**		
	OR	95 % CI		OR	95 % CI		OR	95 % CI	
**Unadjusted Model**									
**PP** (ref=Non-PP)	1.572	1.492	1.656	1.574	1.487	1.665	1.624	1.533	1.722
**E-PP** (ref=Non-PP)	2.069	1.876	2.283	2.095	1.910	2.297	2.216	1.984	2.474
**Adjusted Model**									
**PP** (ref=Non-PP)	1.220	1.145	1.300	1.232	1.152	1.317	1.240	1.156	1.330
**E-PP** (ref=Non-PP)	1.468	1.308	1.647	1.525	1.349	1.723	1.551	1.365	1.764
Disease-specific									
Hypertension	1.142	1.074	1.216	1.144	1.072	1.221	1.173	1.095	1.256
Diabetes	1.108	1.021	1.202	1.123	1.030	1.225	1.118	1.021	1.224
CHF	1.329	1.155	1.528	1.336	1.153	1.548	1.300	1.115	1.516
IHD	1.057	0.976	1.146	1.037	0.953	1.129	1.044	0.954	1.142
Arrhythmia	1.113	0.910	1.361	1.180	0.953	1.461	1.197	0.959	1.494
Gout	1.809	1.468	2.230	1.649	1.323	2.055	1.689	1.344	2.124
Normal-weight (Ref=Under-W)	1.207	1.052	1.386	1.173	1.016	1.355	1.168	1.006	1.357
Over-weight (Ref=Under-W)	1.268	1.100	1.462	1.238	1.067	1.436	1.231	1.056	1.436
Obese (Ref=Under-W)	1.373	1.193	1.581	1.335	1.152	1.546	1.323	1.136	1.541
Hyper-TG	1.174	1.113	1.239	1.182	1.118	1.25	1.161	1.095	1.231
Lower-HDL-C	1.185	1.103	1.272	1.168	1.083	1.259	1.199	1.107	1.298
Hyper-LDL-C	1.001	1.000	1.001	1.000	1.000	1.001	1.000	1.000	1.001
Medication-specific									
ACEI	1.370	1.200	1.564	1.344	1.169	1.545	1.290	1.116	1.492
ARB	1.434	1.346	1.528	1.447	1.353	1.546	1.428	1.331	1.531
Metformin	0.992	0.896	1.099	1.013	0.910	1.128	1.022	0.913	1.144
Statins	0.974	0.911	1.042	0.973	0.906	1.044	0.966	0.896	1.040
NSAIDS	1.049	0.981	1.121	1.054	0.983	1.131	1.060	0.985	1.141
PPI	1.121	1.004	1.251	1.140	1.016	1.280	1.160	1.027	1.311
Allopurinol	1.119	0.652	1.921	1.268	0.721	2.231	1.421	0.781	2.588
Lifestyle-related									
Smoking	1.078	1.003	1.158	1.115	1.032	1.204	1.122	1.036	1.215
Drinking	0.966	0.906	1.029	0.957	0.894	1.024	0.970	0.904	1.041
Physical activity	1.008	0.957	1.062	1.003	0.949	1.060	1.010	0.953	1.070

Non-PP: Non-polypharmacy for daily counts of less than 5 drugs per year; PP: Polypharmacy for daily counts of 5-10 drugs per year; E-PP: Excessive polypharmacy for daily counts of 10 or more drugs per year.
HTN: hypertension; DM: diabetes mellitus; CHF: congestive heart failure; IHD: ischemic heart disease; Underweight: BMI < 18.5; Normal: BMI < 23; Overweight: BMI < 25; Obese: BMI ≥ 25
ACEIs: Angiotensin-Converting-Enzyme Inhibitors; ARBs: Angiotensin II Receptor Blockers; NSAIDs: Non-Steroidal Anti-Inflammatory Drugs; PPIs: Proton Pump Inhibitors

Supplementary Table S3.

Exposure to other medicines related with kidney function

	Matched Case (n=14,577)		Matched Control (n=14,577)		p-value
	Freq.	(%)	Freq.	(%)	
Other medicines related with Kidney function					
Osmotic agent	263	1.80	306	2.10	0.07
Contrast	7	0.05	20	0.14	0.01
Calcineurin inhibitor*	3	0.02	0	0	0.25
Methotrexate	0	0	0	0	1
Fosfomycin*	0	0	1	0	1
Sulfa	25	0.17	14	0.10	0.08
Penicillin	531	3.64	533	3.66	0.95
Cephalosporin	621	4.26	554	3.80	0.05

*Statistical difference was determined on the results of Fisher's exact test.
